# Annexin A1 Mitigates Blood–Brain Barrier Disruption in a Sepsis‐Associated Encephalopathy Model by Enhancing the Expression of Occludin and Zonula Occludens‐1 (ZO‐1)

**DOI:** 10.1111/cns.70173

**Published:** 2024-12-27

**Authors:** Yao Li, Fang Zhou, Jiyue You, Xinran Gong

**Affiliations:** ^1^ Department of Anesthesiology, Sichuan Provincial People's Hospital, School of Medicine University of Electronic Science and Technology of China Chengdu China

**Keywords:** annexin A1, blood brain barrier, occludin, sepsis‐associated encephalopathy, VEGF

## Abstract

**Aims:**

This study investigated the protective role of Annexin A1 (ANXA1) in sepsis‐associated encephalopathy (SAE) by examining its effects on brain vascular endothelium and blood–brain barrier (BBB) integrity.

**Methods:**

Mice were divided into four groups: wild type (WT), cecal ligation and puncture (CLP), ANXA1 knockout (ANXA1[−/−]), and ANXA1(−/−) with CLP. Neurobehavioral changes were assessed using the Y‐maze test, while BBB integrity was evaluated through Evans blue dye (EBD) staining and permeability tests with fluorescein isothiocyanate (FITC)‐dextran.

**Results:**

Results showed that ANXA1 levels were reduced in septic mice, and its deficiency exacerbated cognitive impairment and survival rate reduction. ANXA1 deficiency also upregulated proinflammatory cytokines and adhesion molecules, worsened BBB impairment, and altered expression of tight junction proteins and VEGF‐A/VEGF‐R2. In vitro, ANXA1 Ac2‐26 prevented LPS‐induced increased permeability in bEnd.3 cells by restoring tight junction proteins and reducing VEGF‐A/VEGF‐R2 expression. Notably, VEGF‐A negated the protective effects of ANXA1 Ac2‐26.

**Conclusion:**

The study concludes that ANXA1 reduces BBB permeability to protect against sepsis‐induced brain dysfunction via VEGF‐A/VEGF‐R2 regulation of tight junction proteins, suggesting ANXA1 as a potential therapeutic for SAE.

## Graphical abstract

Reduced expression of ANXA1 was observed in the brain cortex of septic mice. ANXA1 deficiency led to a decreased 7‐day survival rate and exacerbated neurobehavioral changes induced by CLP. Furthermore, ANXA1 deficiency worsened BBB dysfunction in septic mice, with this effect being mediated through occludin and ZO‐1. The protective effects of ANXA1 are mediated by the VEGF‐A/VEGF‐R2 signaling pathway.

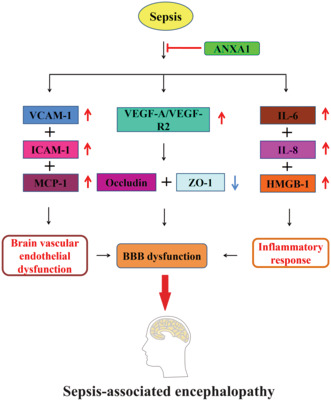

## 1 Introduction

Sepsis is characterized by a dysregulated host response to infection, leading to life‐threatening organ dysfunction. It affects over 30 million people globally each year and is a leading cause of death among critically ill patients. The incidence of sepsis among all hospitalized patients ranges from 1% to 2%, making its treatment one of the most costly for any disease [1, 2]. Despite treatment, patients frequently experience long‐term complications from sepsis, encompassing physiological, psychological, and cognitive impairments [3]. During the acute phase of sepsis, sepsis‐associated encephalopathy (SAE) frequently occurs and is one of the most common complications during both the acute and late stages of the disease. It is strongly associated with increased mortality rates in sepsis patients. In the chronic phase of SAE, over 50% of sepsis survivors experience long‐term, severe cognitive deficits, significantly reducing their quality of life [4, 5].

The potential pathological mechanisms of SAE primarily include cytokine activity, disruption of the blood–brain barrier (BBB), microscopic brain injury, alterations in cerebral microcirculation and metabolism, and changes in neurotransmitter transmission, with multiple mechanisms likely acting together [6]. During sepsis, a systemic inflammatory response syndrome (SIRS) is activated, leading to the release of a large amount of inflammatory mediators such as cytokines and chemokines into the bloodstream [7]. These mediators compromise the integrity of the BBB directly or indirectly, resulting in its dysfunction [8]. Once the BBB is disrupted, harmful substances and immune cells that were previously unable to pass through enter the brain, leading to pathological changes such as brain edema, inflammatory cell infiltration, oxidative stress, and apoptosis. These changes result in brain dysfunction and cognitive impairment [9]. Furthermore, the destruction of the BBB disrupts the brain's original homeostasis, affecting the balance of neurotransmitters and exacerbating neuroinflammation and neurodegenerative changes. Barichello et al. reported that the disruption of the BBB allows peripheral immune cells to infiltrate the brain. This infiltration can trigger or intensify neuroinflammation, which in turn contributes to the neurological symptoms characteristic of SAE [10]. Recent studies have underscored the significance of non‐coding RNAs (ncRNAs), including long noncoding RNAs (lncRNAs), microRNAs (miRNAs), and circular RNAs (circRNAs), in regulating BBB function during the development of SAE [9, 11]. Moreover, the review by Gao et al. highlights that targeting the modulation of microglial activation, addressing endothelial cell dysfunction, and preventing BBB permeability are therapeutic strategies that could substantially influence the outcomes of SAE. These insights suggest that interventions aimed at preserving BBB integrity and function could be pivotal in managing and potentially mitigating the effects of SAE [9]. Therefore, protecting and repairing the integrity of the BBB is of great significance for treating SAE [12].

Zonula Occludens‐1 (ZO‐1) is a crucial tight junction protein integral to the BBB, belonging to the membrane‐associated guanylate kinase (MAGUK) family. It facilitates the formation of tight junctions between endothelial cells, vital for preserving the selective permeability of the BBB [13]. ZO‐1 interacts with various transmembrane proteins, including occludin, claudins, and junctional adhesion molecules (JAMs), as well as cytoplasmic proteins such as actin‐binding proteins and Ras target AF6/afadin. This interaction network helps regulate paracellular permeability and maintain the integrity of the endothelial cell layer that constitutes the BBB [14, 15]. Research has shown that the expression levels of ZO‐1 and occludin can indicate BBB integrity. In conditions like ischemic stroke, modulating ZO‐1 expression through epigenetic modifications can influence BBB repair and neurological function [13]. The role of ZO‐1 extends beyond structural support to include involvement in cellular signaling and response to stress, making it an important factor in neuroprotection and the maintenance of neurological function. Additionally, ZO‐1 has been reported to be associated with the severity of sepsis and the number of organ failures [16]. Vascular endothelial growth factor (VEGF) is a potent growth factor crucial for vasculogenesis and angiogenesis. In the context of the BBB, VEGF has a complex and dual role. It is expressed in neurons, astrocytes, macrophages, and vascular endothelial cells in ischemic and hypoxic brain tissues after cerebral ischemia. While VEGF is essential for angiogenesis, neurogenesis, and neuroprotection, it can also increase BBB permeability and induce endothelial barrier disruption in the early stages of cerebral ischemia [17].

Annexin A1 (ANXA1), located on human chromosome 9q21.13, comprises a *C*‐terminal core domain and a unique *N*‐terminal domain containing 346 amino acids, with a molecular weight of 37 kDa. It is the first member of the Annexins family to be discovered and was initially identified as an inhibitor of phospholipase A2 during studies on the inhibition of leukocyte aggregation in inflammation models [18, 19]. ANXA1 is primarily distributed in the cytoplasm and, upon cell activation, is mobilized to the cell surface [20]. While ANXA1 is abundant in neutrophils, subsequent studies have demonstrated its expression in various tissues, implicating its role in diverse cellular processes and its association with inflammation, cancer, and neurological dysfunction [21, 22, 23]. ANXA1 is highly expressed at the site of cell–cell contacts, specifically at tight junctions, and has been shown to play a key role in maintaining the tightness of the BBB. ANXA1 knockout mice exhibit increased BBB permeability, as measured by MRI, increased leakage of Evans blue extravasation, and elevated serum IgG levels [24]. However, the pharmacological function of ANXA1 in SAE is less reported. In this research, we aim to explore the potential therapeutic effects of ANXA1 in treating SAE and elucidate its underlying mechanisms of action. We will investigate how ANXA1 interacts with various cellular components and signaling pathways to mitigate brain inflammation and reduce BBB disruption by conducting both in vitro and in vivo experiments.

## 2 Materials and Methods

### 2.1 Animal Experimental Procedure and CLP Surgery

C57BL/6 wide‐type mice were obtained from Vital River (Beijing, China), and ANXA1−/− mice were purchased from Charles River Laboratories UK Ltd. (UK), with an average age of 7–9 weeks. CLP surgery was utilized to construct the sepsis model [25].

Mice were fasted and deprived of water for 8 h prior to surgery. After being weighed, they were anesthetized with 2% pentobarbital sodium (4 mL/kg) via intraperitoneal injection. The abdominal skin was shaved and disinfected. Under a laminar flow hood, an incision approximately 2 cm in length was made along the anterior midline of the abdominal wall. The cecum and mesentery were freed, and the base of the cecum was ligated with a No. 5 silk suture in a circular fashion. Once the ligation was completed, it was carefully inspected to ensure that the intestinal passage remained unobstructed. About 1 cm from the end of the cecum, it was punctured twice with a No. 22 needle. A small amount of feces was squeezed out through the puncture holes, wiped clean, and the cecum was returned to its original position. The abdominal wall was then sutured closed layer by layer. Following the surgery, 20 mL/kg of physiological saline was administered subcutaneously for fluid resuscitation. Mice in the Sham group did not undergo ligation or puncture. After the surgery, the mice were housed separately and closely observed for 6 h. The successful establishment of the sepsis model was determined by the presence of shivering, piloerection, lethargy, increased ocular discharge, and diminished protective reflexes within 6 h after the CLP procedure.

For the animal experiments, mice were divided into four groups (20 mice per group): Wild‐type (WT), CLP, ANXA1(−/−), and ANXA1(−/−) + CLP. In the WT and ANXA1(−/−) groups, C57BL/6 WT mice and ANXA1−/− mice underwent sham operations, respectively. In the CLP and ANXA1(−/−) + CLP groups, C57BL/6 WT mice and ANXA1−/− mice underwent CLP operations, respectively.

### 2.2 Survival Rate

Twenty mice were randomly selected from each group and conventionally raised. The survival rates of the mice were observed and recorded on postoperative days 0, 1, 2, 3, 4, 5, 6, and 7.

### 2.3 Y‐Maze Test

The working memory ability of the mice was assessed using the Y‐maze test [26]. The mice were trained daily for 3 days prior to the surgical procedure. The Y‐maze experiment was conducted on days 3, 5, and 7 following the procedure. The Y‐maze consisted of three arms, each forming an angle of 120° with the others. The arms were randomly designated as Arm A, Arm B, and Arm C. Each mouse was placed at the start of Arm A and allowed to move freely for 5 min. The movement of the mice between arms was recorded using the Any‐maze video analysis system. Based on the video recordings, the total number of entries into each arm and the number of spontaneous alternations made by the mice were recorded. The percentage of spontaneous alternations was calculated to evaluate the working memory ability of the mice, and the total number of alternations was used to assess the activity level. The percentage of spontaneous alternations was calculated as:

$$\begin{array}{c} \text{Percentage of spontaneous alternations}=\\ \left(\text{the number of spontaneous alternations}\times 100\right)/\\ \left(\text{the total number of alternations}-2\right) \end{array}$$

### 2.4 Evans Blue Dye (EBD) Staining

Mice were intravenously injected through the tail vein with 0.5% EB (Cat# E2129; Sigma‐Aldrich, USA) at a dose of 0.1 mL per 10 g. After circulating for 1 h, the mice were euthanized by transcardial perfusion with 0.9% saline until the fluid exiting the right atrium was clear. Brain tissue was then harvested, and 1 mL of PBS was added. The tissue was quickly homogenized and centrifuged at 1000 × *g* for 15 min. The supernatant was collected, and absorbance was measured using a microplate reader (MD, USA) at a wavelength of 632 nm. The amount of EB extravasation was determined based on the standard curve [27].

### 2.5 Cell Culture and Treatment

bEnd.3 brain microvascular endothelial cells (BMVECs) were purchased from ATCC (USA) and cultured in complete endothelial culture medium (ECM, Cat#211–500; MERK, USA) supplemented with 10% fetal bovine serum (FBS; Cat# A5670401; ThermoFisher Scientific, USA), 100 units/mL of penicillin, and 100 μg/mL of streptomycin (Cat# CL12331; ChemeGen, Shanghai, China) under 5% CO₂ at 37°C [28]. All experiments were carried out when cell confluence reached 90%.

### 2.6 Fluorescein Isothiocyanate (FITC)‐Dextran Experiments

bEnd.3 cells were seeded onto the filter membrane of the chamber at a density of 2 × 10^5^ cells/cm^2^. After the bEnd.3 cells formed a confluent monolayer, the culture medium in the chamber was discarded. In the lower chamber, 600 μL of serum‐free ECM without FITC‐labeled dextran (Cat# ST2930; Beyotime Biotechnology, Shanghai, China) was added, and in the upper chamber, 100 μL of 0.5 mg/mL FITC‐dextran prepared with serum‐free ECM was added. After a 1‐h incubation period, the solutions from both the upper and lower chambers were aspirated in a dark room, 100 μL per well, and the OD values (485/520 nm) were measured using a multifunctional microplate reader (MD, USA) [29].

### 2.7 Real Time PCR Assay

Cell or cortex tissue samples were collected, and RNA was extracted from the cells using Trizol (Cat# 15596018CN; Invitrogen, USA), chloroform, isopropanol, and 75% ethanol (by volume fraction). The RNA concentration was measured, and its integrity was assessed by agarose gel electrophoresis. Reverse transcription was performed using an RNA reverse transcription kit (Roche, Switzerland), and the resulting cDNA was stored at −80°C. For subsequent fluorescence quantitative detection of mRNA, the cDNA samples were diluted with RNase‐free water in a 1:1 ratio before the reaction. The PCR amplification conditions were as follows: initial denaturation at 95°C for 10 min (1 cycle), denaturation at 95°C for 15 s, and annealing/extension at 60°C for 60 s for 40 cycles.

### 2.8 Western Blotting Assay

Cortex tissue samples weighing approximately 0.1 g or cells were lysed on ice using RIPA lysis buffer. After centrifugation at 4°C, the supernatant was collected. Protein concentration was determined using the bicinchoninic acid (BCA) assay, and 30 μg of total protein was loaded for electrophoresis. Following sodium dodecyl sulfate‐polyacrylamide gel electrophoresis (SDS‐PAGE), proteins were transferred onto a polyvinylidene fluoride (PVDF) membrane. The membrane was blocked with 5% non‐fat milk for 2 h and then incubated overnight at 4°C with primary antibodies against ANXA1 (Cat# 30797, 1:1000; CST, USA), occludin (Cat# 91131, 1:500; CST), ZO‐1 (Cat# 13663, 1:1000; CST), VEGF‐R2 (Cat# 2479, 1:1000; CST), and β‐actin (Cat# 4970, 1:5000; CST). After washing the membrane with TBS containing Tween‐20 (TBST), it was incubated with the horseradish peroxidase (HRP)‐conjugated secondary antibody (Cat# 7074, 1:4000; CST) for 2 h. The membrane was then developed using an electrochemiluminescence (ECL) reagent for luminescence detection, and the grayscale values of each protein band were measured using image analysis software. The relative expression level of the target protein was calculated as the ratio of the grayscale value of the target protein to that of the internal control (β‐actin) [28].

### 2.9 Enzyme‐Linked Immunosorbent Assay (ELISA)

Commercial kits for interleukin‐6 (IL‐6) (Cat# ab178013; Abcam, USA), interleukin‐8 (IL‐8) (Cat# ab214030; Abcam), high mobility group box‐1 (HMGB‐1) (Cat# abx151824, Abbexa, Beijing, China), vascular cellular adhesion molecule‐1 (VCAM‐1) (Cat# ab223591; Abcam), intercellular adhesion molecule‐1 (ICAM‐1) (Cat# ab314854; Abcam), monocyte chemotactic protein‐1 (MCP‐1) (Cat# ab179886; Abcam), and vascular endothelial growth factor A (VEGF‐A) (Cat# ab119566; Abcam) were used to detect protein secretion levels, following the manufacturer's instructions strictly. Briefly, 1 g of cortex tissue was weighed and homogenized at 14,000 × g for 15 min at 4°C. The supernatant was collected for detection. Fifty microliters (50 μL) of each sample or standard were added into pre‐coated 96‐well plates, in triplicate, followed by the addition of 50 μL of antibody cocktail and incubation for 1 h. Each well was washed three times with 350 μL of 1× Wash Buffer. Then, 100 μL of TMB Development Solution was added to each well and incubated for 10 min in the dark on a plate shaker set to 400 rpm. Afterward, 100 μL of stop solution was added, and the plate was shaken for 1 min, followed by recording the optical density (OD) at 450 nm.

### 2.10 Immunostaining Assay

Cortex tissues were fixed in 4% paraformaldehyde for 24 h, followed by embedding in wax and sectioning into 5 μm slices. After dewaxing, hydration, and antigen retrieval, the slices were incubated with primary antibodies against occludin, ZO‐1, VEGF‐A, or VEGF‐R2 (1:200; CST, USA) overnight at 4°C. The slices were then incubated with the Alexa Fluor 488 or Alexa Fluor 647 secondary antibody working solution (1:400; CST) for 1 h. After incubation, the slices were mounted, observed, and photographed under a fluorescent microscope (Zeiss, Germany). The average optical density of positive staining within a unit area was measured using Image Pro‐Plus 6.0 software [30].

### 2.11 Statistical Analysis

The statistical analysis was performed using GraphPad Prism 9 software. To assess the normality of continuous variables, a Kolmogorov–Smirnov test was employed, and Levene's test was used to evaluate the homogeneity of variance. For data with a normal distribution (mean ± standard deviation), comparisons between two groups were made using a *t*‐test, while multiple group comparisons were analyzed through ANOVA followed by Scheffe's post hoc test. Data that did not follow a normal distribution in multiple group comparisons were analyzed using a Kruskal‐Wallis test, with subsequent pairwise comparisons conducted via Dunn's post hoc test. A *p*‐value < 0.05 was considered statistically significant.

## 3 Results

### 3.1 Reduced Levels of ANXA1 Were Observed in the Cortex Tissues of Septic Mice

**FIGURE 1:**
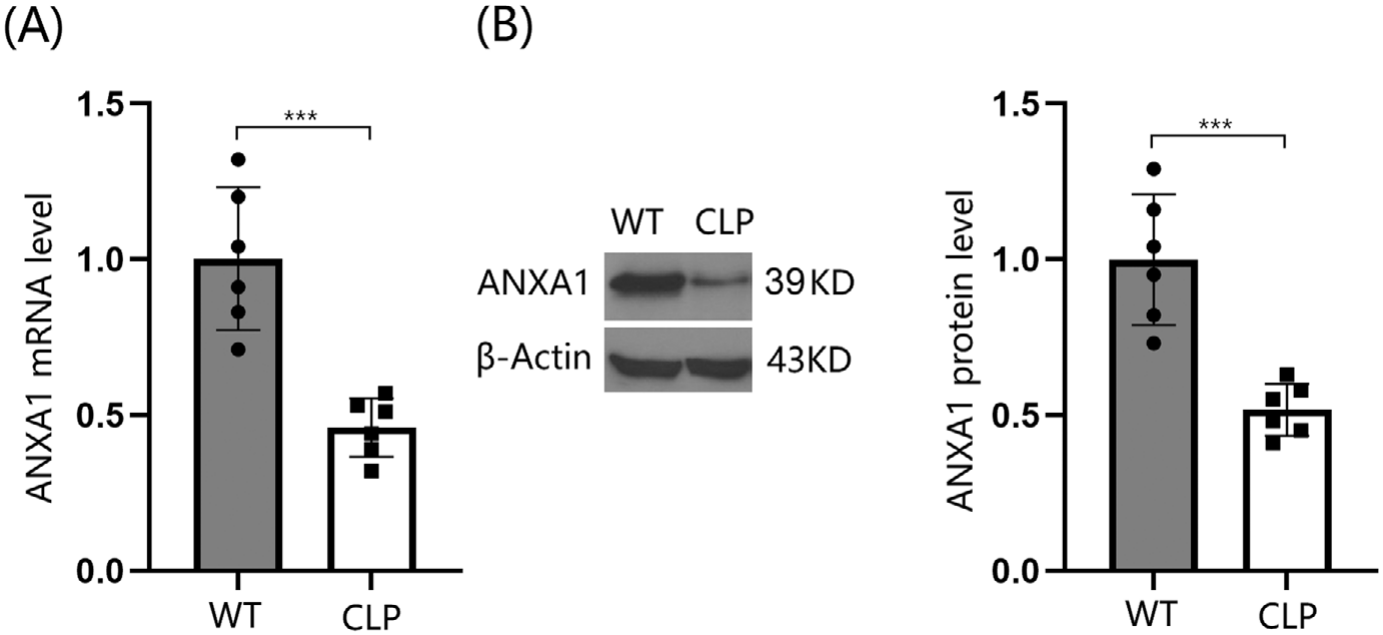
Reduced expression of Annexin A1 (ANXA1) was observed in the brain cortex of septic mice. The septic mice model was established using cecal ligation and puncture (CLP). (A) ANXA1 mRNA levels in the cortex; (B) ANXA1 protein levels (***p < 0.005 vs. WT group).

Sepsis was induced in mice using the CLP model, and the expression of ANXA1 was assessed. Notably, ANXA1 was significantly downregulated in the brain cortex tissues of these septic mice (Figure 1A,B), (Supporting Information Data S1) suggesting a potential beneficial role for ANXA1 in the treatment of SAE.

### 3.2 ANXA1 Deficiency Reduced the 7‐Day Survival Rate and Exacerbated CLP‐Induced Neurobehavioral Changes in Septic Mice

**FIGURE 2:**
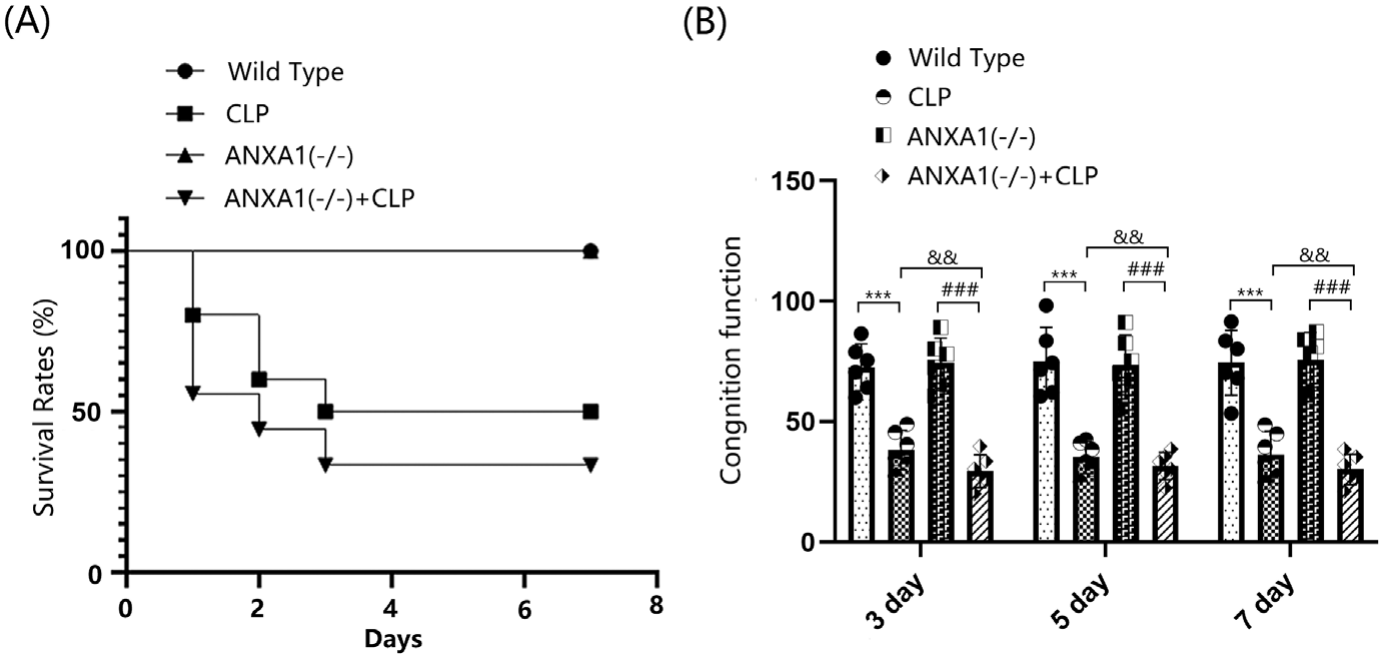
Annexin A1 (ANXA1) deficiency reduced the 7‐day survival rate and exacerbated CLP‐induced neurobehavioral changes in septic mice. Mice were divided into four groups: WT, CLP, ANXA1 (−/−), and ANXA1 (−/−) + CLP. (A) The survival rate was analyzed at day 0, 1, 2, 3, 4, 5, 6, and 7 post‐operation; (B) cognitive function at days 3, 5, and 7 after the operation was assessed using the Y‐maze test (***p < 0.005 vs. WT group; ^###^ *p* < 0.005 vs. ANXA1 (−/−) group; ^&&^ *p* < 0.01 vs. CLP group).

To evaluate the role of ANXA1 in SAE, mice were divided into four groups: WT, CLP, ANXA1(−/−), and ANXA1(−/−) + CLP. The survival rate in the WT and ANXA1(−/−) groups remained at 100% from day 0 to day 7 post‐surgery. In contrast, the survival rate in the CLP group sharply decreased from 100% to 50% between day 0 and day 3 post‐surgery, and remained at 50% from day 4 to day 7. The survival rate in the ANXA1(−/−) + CLP group significantly decreased from 100% to 30% between day 0 and day 3, and remained at 30% from day 4 to day 7. Compared to the CLP group, the stable survival rate was notably reduced from 50% to 30% after ANXA1 silencing (Figure 2A). In the Y‐maze test, the percentage of spontaneous alternations on days 3, 5, and 7 post‐surgery was significantly reduced in both WT and ANXA1(−/−) mice following the CLP operation. Moreover, compared to the CLP group, the percentage of spontaneous alternations on days 3, 5, and 7 post‐surgery in the ANXA1(−/−) + CLP group was significantly lower (Figure 2B).

### 3.3 ANXA1 Deficiency Exacerbated Inflammation in the Cortex Tissues of Septic Mice

**FIGURE 3:**
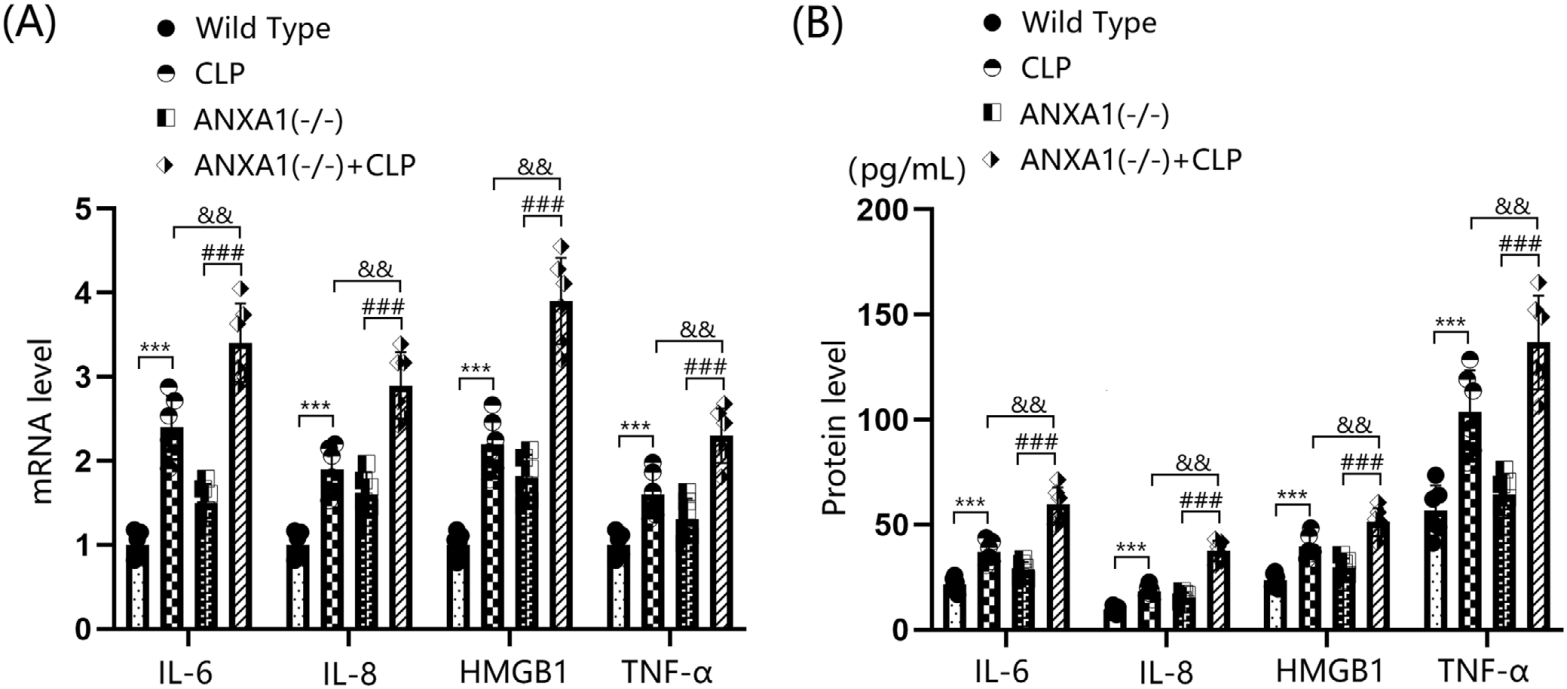
ANXA1 deficiency exacerbated the expression of inflammatory cytokines IL‐6, IL‐8, HMGB1, and TNF‐α in the brain cortex of septic mice. Mice were divided into four groups: WT, CLP, ANXA1 (−/−), and ANXA1 (−/−) + CLP. (A) MRNA levels of IL‐6, IL‐8, HMGB1, and TNF‐α in the cortex; (B) protein levels of IL‐6, IL‐8, HMGB1, and TNF‐α in the cortex (***p < 0.005 vs. WT group; ^###^ *p* < 0.005 vs. ANXA1 (−/−) group; ^&&^ *p* < 0.01 vs. CLP group).

The gene expression levels of IL‐6, IL‐8, HMGB‐1, and TNF‐α in the cortex tissues of both WT and ANXA1(−/−) mice were significantly upregulated by the CLP operation. Furthermore, in CLP‐operated mice, the gene expression levels of IL‐6, IL‐8, HMGB‐1, and TNF‐α in the cortex tissues were notably increased by silencing ANXA1 (Figure 3A). IL‐6 protein levels were markedly increased from 21.6 to 37.1 pg/mL following the CLP operation, and from 21.6 to 28.6 pg/mL after ANXA1 silencing. Compared to the CLP group, IL‐6 levels were significantly elevated to 59.5 pg/mL in the ANXA1(−/−) + CLP group. IL‐8 concentrations in the WT, CLP, ANXA1(−/−), and ANXA1(−/−) + CLP groups were 9.5, 18.3, 15.3, and 37.5 pg/mL, respectively. HMGB‐1 levels were noticeably increased from 23.5 to 39.6 pg/mL following the CLP operation, and from 23.5 to 29.5 pg/mL after ANXA1 silencing. Compared to the CLP group, HMGB‐1 levels were strikingly elevated to 51.3 pg/mL in the ANXA1(−/−) + CLP group (Figure 3B). Additionally, TNF‐α concentrations in the WT, CLP, ANXA1(−/−), and ANXA1(−/−) + CLP groups were 56.8, 103.7, 64.3, and 136.7 pg/mL, respectively.

### 3.4 ANXA1 Deficiency Exacerbated the Expression of Vascular Endothelial Dysfunction Biomarkers in the Cortex Tissues of Septic Mice

**FIGURE 4:**
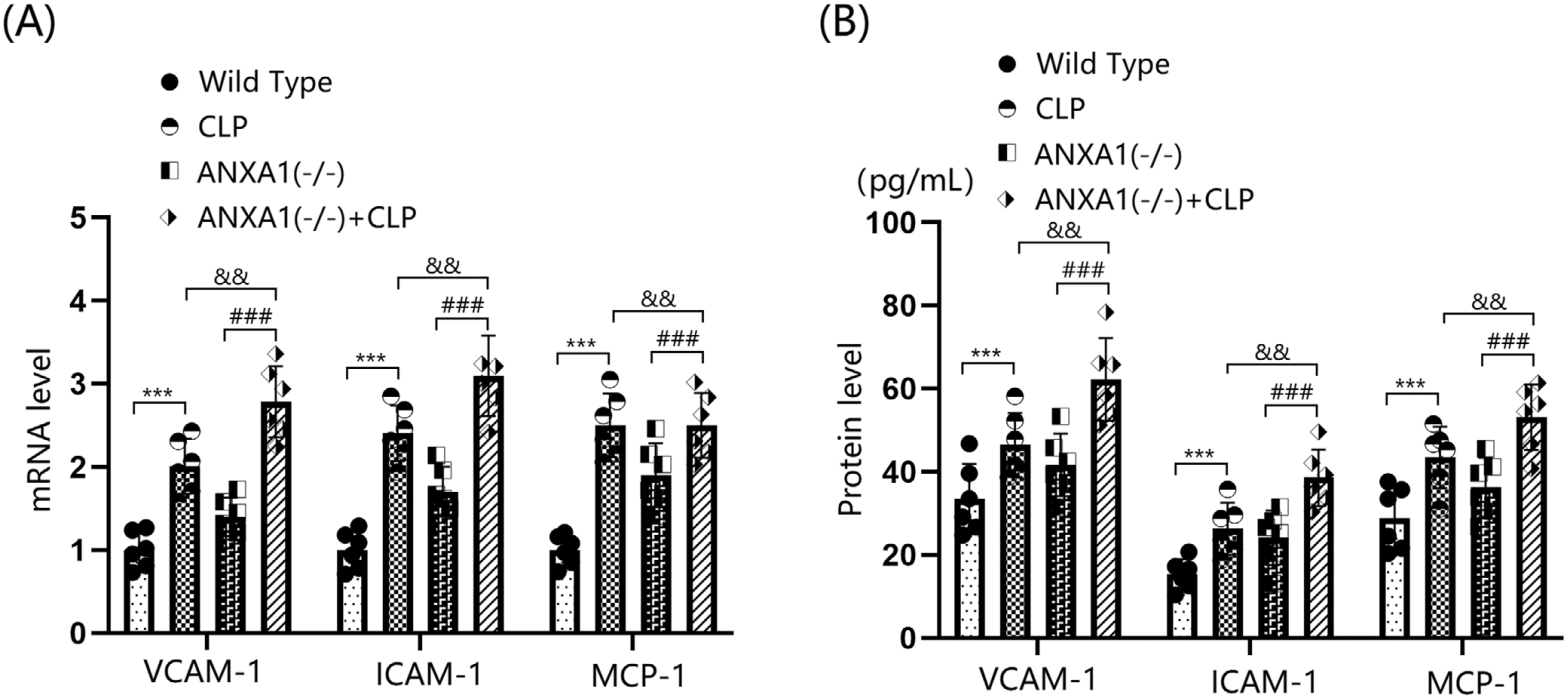
ANXA1 deficiency aggravated the expression of biomarkers of vascular endothelial dysfunction in the brain cortex of septic mice. Mice were divided into four groups: WT, CLP, ANXA1 (−/−), and ANXA1 (−/−) + CLP. (A) MRNA levels of VCAM‐1, ICAM‐1, and MCP‐1 in the cortex; (B) protein levels of VCAM‐1, ICAM‐1, and MCP‐1 in the cortex (***p < 0.005 vs. WT group; ^###^ *p* < 0.005 vs. ANXA1 (−/−) group; ^&&^ *p* < 0.01 vs. CLP group).

Brain vascular endothelial dysfunction is a critical risk factor for BBB disruption [31]. In this study, the levels of VCAM‐1, ICAM‐1, and MCP‐1 in the cortex tissues of both WT and ANXA1(−/−) mice were sharply upregulated by the CLP operation. In CLP‐operated mice, the levels of VCAM‐1, ICAM‐1, and MCP‐1 in the cortex tissues were markedly increased by silencing ANXA1 (Figure 4A). VCAM‐1 levels in the WT, CLP, ANXA1(−/−), and ANXA1(−/−) + CLP groups were 33.4, 46.5, 41.3, and 62.3 pg/mL, respectively. ICAM‐1 production was noticeably increased from 15.3 to 26.3 pg/mL following the CLP operation, and from 15.3 to 24.2 pg/mL after ANXA1 silencing. Compared to the CLP group, ICAM‐1 levels were significantly elevated to 38.6 pg/mL in the ANXA1(−/−) + CLP group. Furthermore, MCP‐1 concentrations in the WT, CLP, ANXA1(−/−), and ANXA1(−/−) + CLP groups were 28.9, 43.5, 36.3, and 53.2 pg/mL, respectively (Figure 4B).

### 3.5 ANXA1 Deficiency Exacerbated BBB Disruption and the Reduction of Occludin and ZO‐1 in the Cortex Tissues of Septic Mice

**FIGURE 5:**
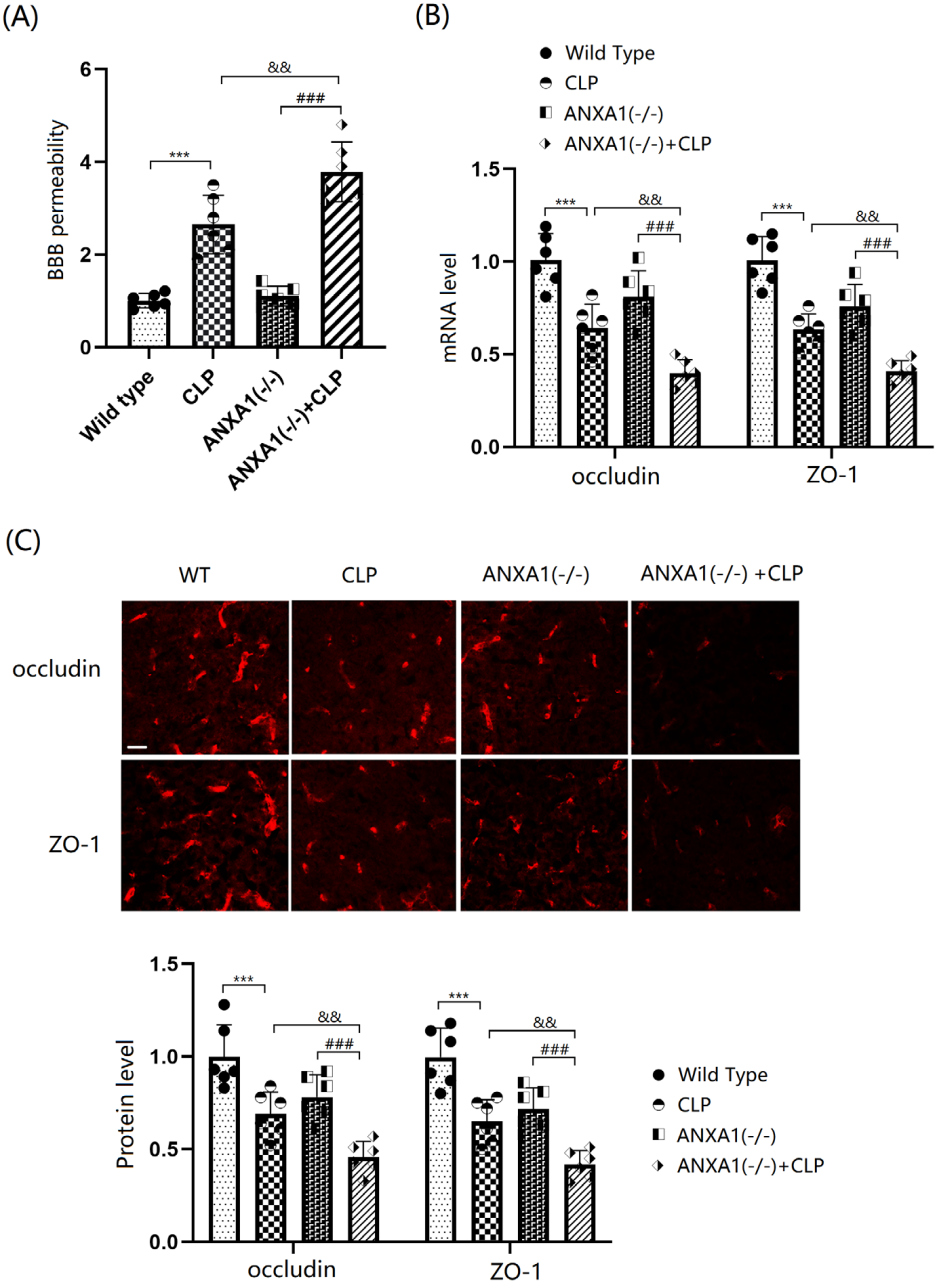
ANXA1 deficiency exacerbated the dysfunction of BBB integrity and reduction of occludin and ZO‐1 in the brain cortex of septic mice. Mice were divided into four groups: WT, CLP, ANXA1 (−/−), and ANXA1 (−/−) + CLP. (A) BBB permeability was measured using EBD staining; (B) mRNA levels of occludin and ZO‐1; (C) protein levels of occludin and ZO‐1 as measured by immunostaining. Scale bar, 100 μm (***p < 0.005 vs. WT group; ^###^ *p* < 0.005 vs. ANXA1 (−/−) group; ^&&^ *p* < 0.01 vs. CLP group).

BBB disruption was assessed using the EB dye extravasation assay. The results indicate that BBB permeability in WT mice was significantly increased by the CLP operation, while only a slight change was observed in ANXA1(−/−) mice. In CLP‐operated mice, BBB permeability was sharply increased by ANXA1 silencing (Figure 5A). Levels of occludin and ZO‐1 in the cortex tissues of both WT and ANXA1(−/−) mice were markedly reduced by the CLP operation. In CLP‐operated mice, the levels of occludin and ZO‐1 were notably further reduced by silencing ANXA1 (Figure 5B,C).

### 3.6 ANXA1 Deficiency Exacerbated the Increase in the Expression of VEGF‐A and VEGF‐R2 in the Cortex Tissues of Septic Mice

**FIGURE 6:**
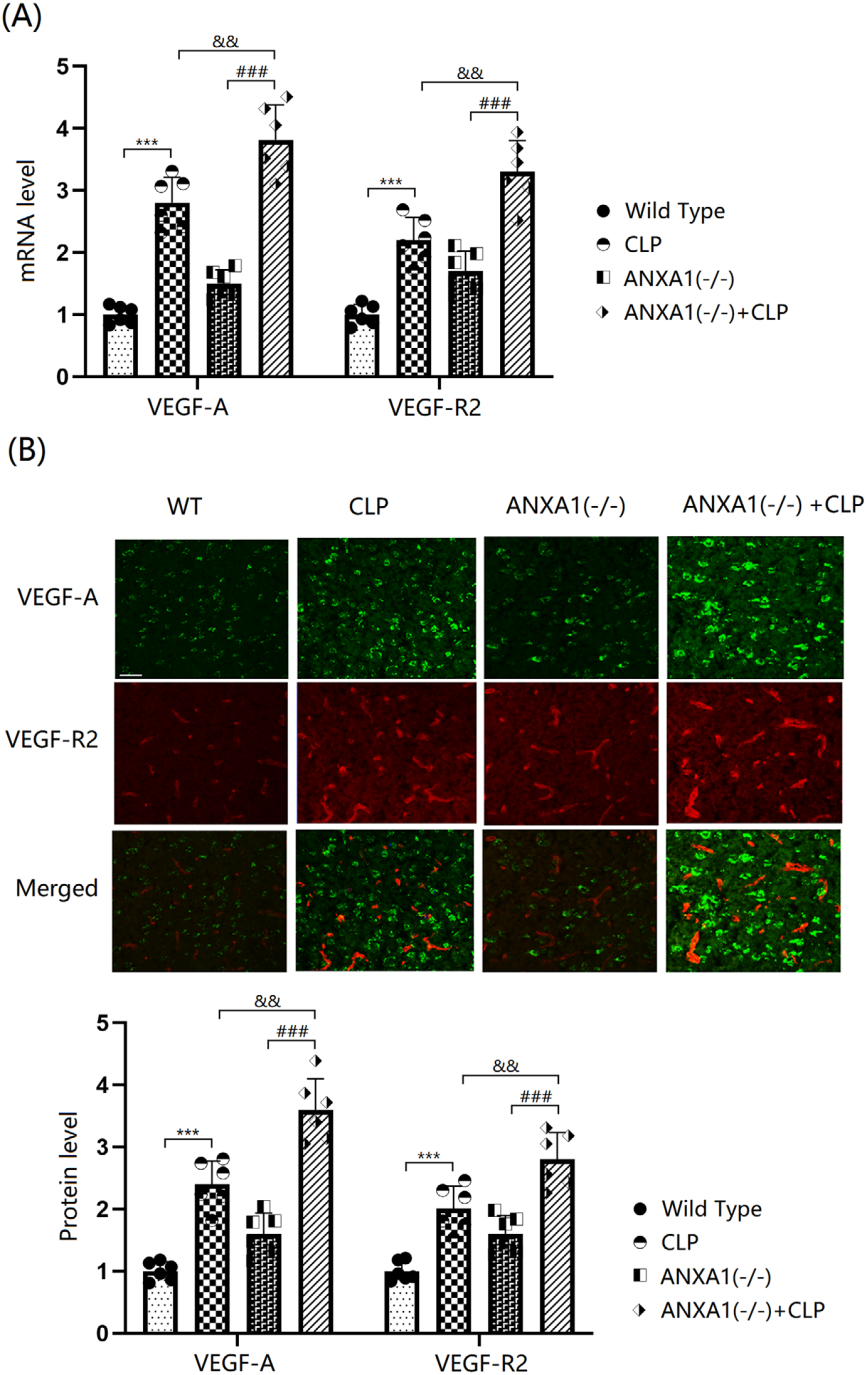
ANXA1 deficiency aggravated the increase in the expression of VEGF‐A and VEGF‐R2 in the brain cortex of septic mice. Mice were divided into four groups: WT, CLP, ANXA1 (−/−), and ANXA1 (−/−) + CLP. (A) mRNA levels of VEGF‐A and VEGF‐R2; (B) protein levels of VEGF‐A and VEGF‐R2. Scale bar, 100 μm (***p < 0.005 vs. WT group; ^###^ *p* < 0.005 vs. ANXA1 (−/−) group; ^&&^ *p* < 0.01 vs. CLP group).

The VEGF‐A/VEGF‐R2 axis is known to play a key role in vascular permeability. In this study, VEGF‐A and VEGF‐R2 levels in the cortex tissues of both WT and ANXA1(−/−) mice were significantly increased by the CLP operation. Moreover, in CLP‐operated mice, VEGF‐A and VEGF‐R2 levels were further enhanced by silencing ANXA1 (Figure 6A,B).

### 3.7 The Peptide Annexin A1 (Ac2‐26) Prevented Lipopolysaccharide (LPS)‐Induced Increases in Brain Endothelial Monolayer Permeability in BEnd.3 BMVECs

**FIGURE 7:**
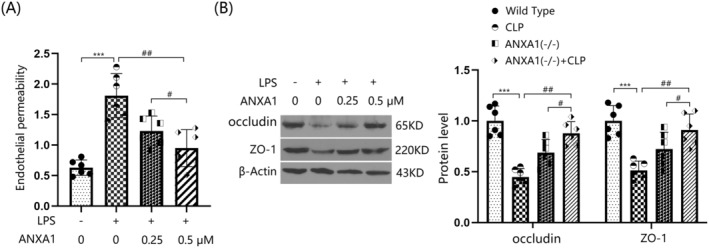
The peptide Annexin A1 (Ac2‐26) prevented LPS‐induced increased brain endothelial monolayer permeability in bEnd.3 brain microvascular endothelial cells (BVECs). Cells were stimulated with LPS (1 μg/mL) with or without ANXA1 (Ac2‐26) (0.25, 0.5 μM) for 48 h. (A) Endothelial permeability was assessed by FITC‐dextran permeation; (B) protein expression of occludin and ZO‐1 (***p < 0.005 vs. control group; ^##^, ^###^ *p* < 0.01, 0.005 vs. LPS group).

bEnd.3 BMVECs were stimulated with LPS (1 μg/mL) with or without ANXA1 (Ac2‐26) (0.25 or 0.5 μM) for 48 h [32]. The concentration of permeated FITC was sharply increased from 0.63 to 1.81 pg/mL by LPS, which was significantly reduced to 1.23 and 0.95 pg/mL by 0.25 and 0.5 μM ANXA1 (Ac2‐26), respectively (Figure 7A). Moreover, occludin and ZO‐1 levels were notably suppressed in LPS‐stimulated bEnd.3 BMVECs, but were markedly enhanced by treatment with 0.25 and 0.5 μM ANXA1 (Ac2‐26) (Figure 7B) (Supporting Information Data S1).

### 3.8 Annexin A1 (Ac2‐26) Reduced the Expression of VEGF‐A and VEGF‐R2 in BEnd.3 BMVECs

**FIGURE 8:**
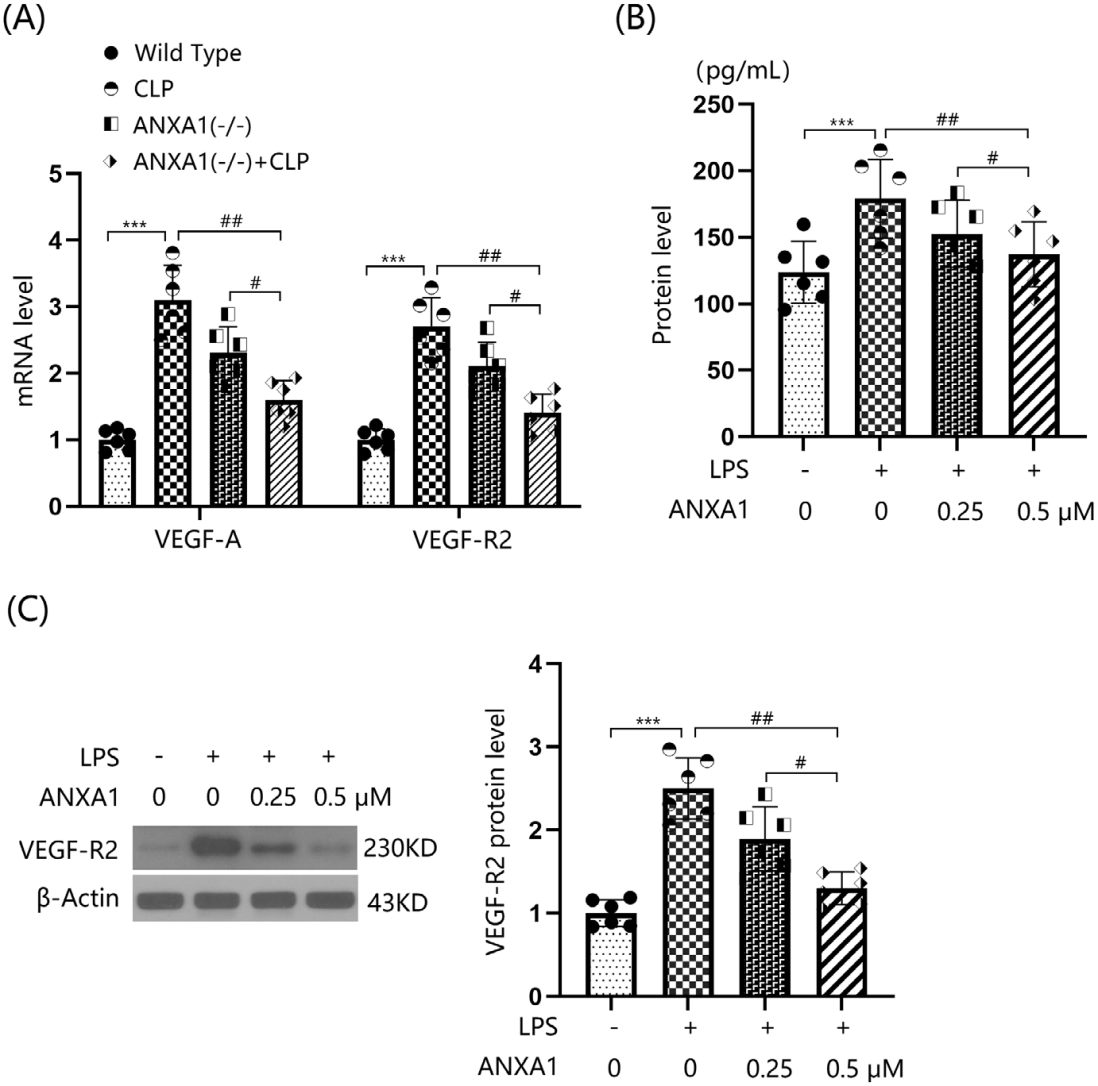
Annexin A1 (Ac2‐26) reduced the expression of VEGF‐A and VEGF‐R2 in bEnd.3 BVECs. Cells were stimulated with LPS (1 μg/mL) with or without ANXA1 (Ac2‐26) (0.25, 0.5 μM) for 48 h. (A) mRNA levels of VEGF‐A and VEGF‐R2; (B) protein levels of VEGF‐A; (C) protein levels of VEGF‐R2 (***p < 0.005 vs. control group; ^##^, ^###^ *p* < 0.01, 0.005 vs. LPS + group).

The gene expression levels of VEGF‐A and VEGF‐R2 in bEnd.3 cells were significantly upregulated by LPS, but were notably repressed by 0.25 and 0.5 μM ANXA1 (Ac2‐26) (Figure 8A). VEGF‐A production in LPS‐stimulated bEnd.3 BMVECs increased from 123.8 to 179.1 pg/mL, but was significantly reduced to 152.3 and 137.2 pg/mL by 0.25 and 0.5 μM ANXA1 (Ac2‐26), respectively (Figure 8B). Moreover, the markedly upregulated VEGF‐R2 levels in LPS‐stimulated bEnd.3 cells were noticeably restrained by 0.25 and 0.5 μM ANXA1 (Ac2‐26) (Figure 8C) (Supporting Information Data S1).

### 3.9 VEGF‐A Abolished the Protective Effects of ANXA1 (Ac2‐26) on LPS‐Induced Increased Brain Endothelial Monolayer Permeability in BEnd.3 BMVECs

**FIGURE 9:**
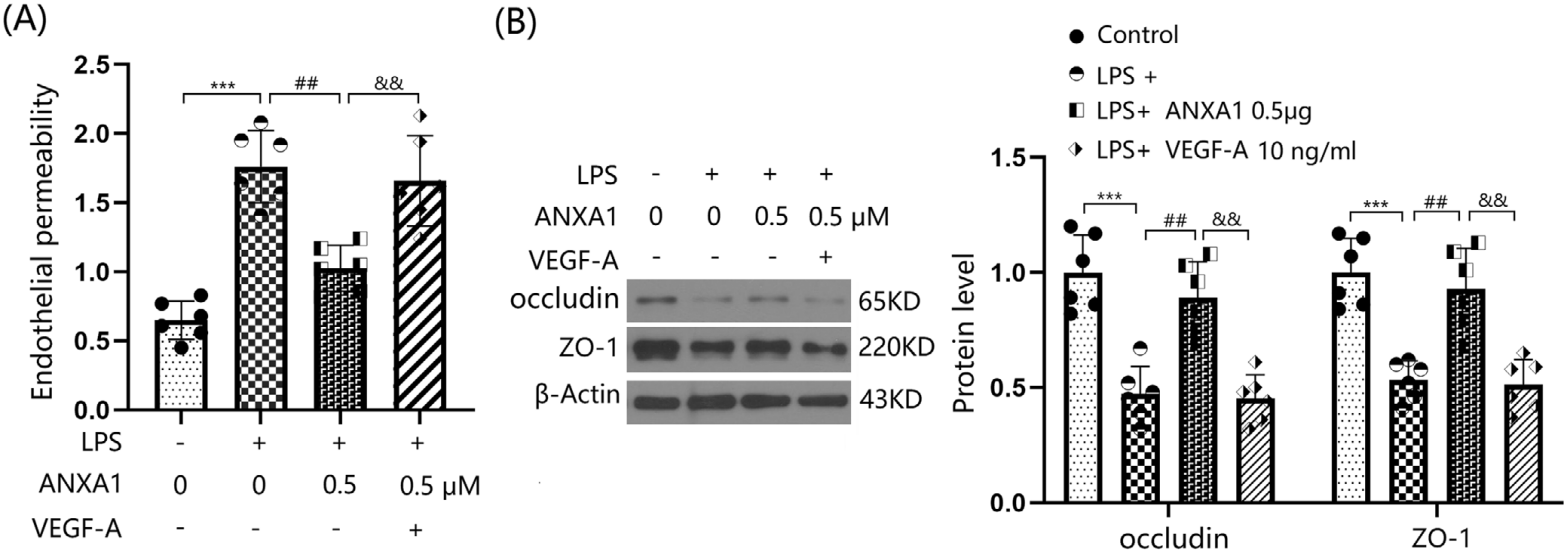
VEGF‐A abolished the protective effects of ANXA1 (Ac2‐26) against LPS‐induced increased brain endothelial monolayer permeability in bEnd.3 BVECs. Cells were stimulated with LPS (1 μg/mL) with or without ANXA1 (Ac2‐26) (0.5 μM) or VEGF‐A (10 ng/mL). (A) Brain endothelial permeability was assessed by FITC‐dextran permeation; (B) protein expression of occludin and ZO‐1 (***p < 0.005 vs. control group; ^##^ *p* < 0.01 vs. LPS group; ^&&^ *p* < 0.01 vs. LPS + ANXA1 group).

To confirm the role of VEGF‐A in ANXA1 (Ac2‐26) function, cells were stimulated with LPS (1 μg/mL) with or without ANXA1 (Ac2‐26) (0.5 μM) or VEGF‐A (10 ng/mL). The concentration of permeated FITC was appreciably elevated from 0.65 to 1.76 pg/mL by LPS, but was strikingly reduced to 1.03 pg/mL by ANXA1 (Ac2‐26). Following the addition of VEGF‐A, the concentration of permeated FITC was notably increased to 1.66 pg/mL (Figure 9A). Furthermore, downregulated occludin and ZO‐1 levels in LPS‐challenged bEnd.3 BMVECs were significantly increased by ANXA1 (Ac2‐26), but were remarkably repressed by VEGF‐A (Figure 9B) (Supporting Information Data S1).

## 4 Discussion

The BBB is composed of endothelial cells (ECs), astrocytes, and pericytes, serving as a highly selective biological interface between the brain parenchyma and the cerebral circulation. The BBB provides essential protection for brain tissue by preventing harmful substances in the peripheral circulation from entering, which is crucial for maintaining the normal function of neurons and the extracellular environment of the central nervous system [33]. During the host response to immune dysregulation induced by LPS, inflammatory cytokines such as IL‐6 and IL‐8 activate endothelial cells (ECs). Once activated, these cells produce reactive oxygen species (ROS), which in turn increase the permeability of the ECs [34]. Additionally, activated ECs can induce the expression and upregulation of adhesion molecules, facilitating the entry of immune cells and pro‐inflammatory factors into the central nervous system through the compromised BBB [35]. In accordance with data presented by Yue et al. [36], following CLP surgery, there was a marked decrease in survival rate, disrupted cognitive function, increased BBB permeability, and aggravated cortical tissue inflammation, all of which were further exacerbated by silencing ANXA1. These findings imply that the loss of ANXA1 may contribute to the development of SAE and BBB disruption.

Furthermore, in agreement with Liu's report [37], the permeability of the bEnd.3 monolayer was significantly increased by LPS, but was notably reduced by ANXA1 (Ac2‐26), an active peptide of ANXA1 [38], indicating the protective effect of ANXA1 on BBB function. ECs are the key cell type that constitute the inner lining of blood vessels and play an essential role in maintaining the integrity of the BBB. ECs form tight intercellular connections such as tight junction (TJ) proteins and adherens junctions, which uphold the physical barrier of the BBB. These connections ensure that large molecules and harmful substances cannot easily pass through the vessel walls [39]. Moreover, ECs regulate the transmembrane transport of specific molecules through transport systems, thereby maintaining the brain's nutrient supply and facilitating the clearance of metabolic waste [40]. The functional state of ECs directly impacts the integrity of the BBB. In pathological conditions such as inflammation, infection, or neurodegenerative diseases, ECs may become damaged or activated, leading to BBB dysfunction. When activated, ECs may increase their permeability, allowing inflammatory cells and cytokines to enter the brain. This can potentially initiate or intensify neuroinflammation and tissue damage [41, 42]. Therefore, maintaining the health and functionality of ECs is essential for protecting the integrity of the BBB. VCAM‐1 and ICAM‐1 are primarily involved in the adhesion process between leukocytes and ECs. This adhesion is a necessary condition for the migration of immune cells to sites of inflammation [43]. MCP‐1, a chemokine, attracts immune cells such as monocytes to move towards areas of inflammation. This process regulates the immune response and inflammatory processes in ECs [44]. In this study, vascular endothelial dysfunction was observed in the cortex tissues of mice following a CLP operation. This dysfunction was significantly exacerbated by silencing ANXA1, suggesting that the loss of ANXA1 might contribute to the endothelial dysfunction during SAE.

Tight junctions (TJs), located at the apical side of BMECs, form a complex of protein molecules crucial for maintaining the integrity of the BBB. These junctions can be categorized into three types based on their cellular localization: transmembrane proteins, cytoplasmic attachment proteins, and cytoskeletal proteins. The transmembrane proteins primarily consist of occludin, claudins, and junctional adhesion molecules, while the cytoplasmic attachment proteins include ZO‐1, AF‐6, and others [45]. Occludin and ZO‐1, in particular, are closely associated with the compromised function of the BBB [46, 47].

In this study, consistent with Li's report [48], TJs were noticeably disrupted by the CLP operation. This disruption was further aggravated by silencing ANXA1, suggesting that the loss of ANXA1 function could contribute to BBB dysfunction through TJ disruption. The protective effect of ANXA1 on TJ proteins was further validated by increased levels of occludin and ZO‐1 observed in Annexin A1 (Ac2‐26) treated bEnd.3 cells. The VEGF‐A/VEGF‐R2 pathway is closely associated with the regulation of endothelial system function [49]. Additionally, the binding of VEGF‐A to VEGF‐R2 activates endothelial nitric oxide synthase, which subsequently leads to decreased expression levels of TJ proteins and disruption of BBB integrity [50, 51]. Herein, the VEGF‐A/VEGF‐R2 axis was activated in mice following the CLP operation, an effect that was further enhanced by silencing ANXA1. Furthermore, the activated VEGF‐A/VEGF‐R2 axis in LPS‐challenged bEnd.3 cells was repressed by ANXA1 (Ac2‐26), implying a significant role of the VEGF‐A/VEGF‐R2 axis in the mechanism involving ANXA1. Moreover, the influence of ANXA1 (Ac2‐26) against LPS‐induced increased brain endothelial monolayer permeability in bEnd.3 cells was abolished by VEGF‐A. This finding validated that ANXA1 protects BBB integrity by repairing VEGF‐A‐mediated TJ disruption.

The results presented indicate that ANXA1 plays a crucial role in maintaining BBB integrity and protecting against sepsis‐induced brain dysfunction. The observation that reduced ANXA1 levels are associated with increased severity of septic conditions suggests that ANXA1 could be a potential biomarker for the progression and severity of sepsis‐related neurological complications. Furthermore, the finding that ANXA1 deficiency exacerbates inflammation, endothelial dysfunction, and BBB disruption highlights the importance of ANXA1 in the pathophysiology of sepsis‐induced brain injury. This suggests that targeting ANXA1 or its pathways could offer new therapeutic strategies for managing SAE. The protective effects of the ANXA1 peptide (Ac2‐26) against LPS‐induced increased permeability in brain endothelial cells and its ability to reduce the expression of VEGF‐A and VEGF‐R2 provide further evidence for the therapeutic potential of ANXA1 modulation. The reversal of these protective effects by VEGF‐A underscores the complex interplay between ANXA1 and VEGF signaling in regulating BBB function. Clinically, these findings support the development of ANXA1‐based therapies for SAE and other conditions involving BBB disruption. They also suggest that monitoring ANXA1 levels could help in assessing the risk of developing SAE and in evaluating the effectiveness of treatments aimed at restoring BBB integrity and reducing neuroinflammation.

The findings of this study, along with their significant clinical implications, chart the course for future research endeavors. First, upcoming investigations should aim to elucidate the precise regulatory mechanisms underlying the interaction between ANXA1 and the VEGF‐A/VEGF‐R2 signaling pathways in brain endothelial cells, particularly focusing on their impact on blood–brain barrier (BBB) integrity during sepsis. Second, there is a need to explore the therapeutic potential of ANXA1‐derived peptides as interventions for sepsis‐induced cognitive impairment. This exploration should encompass dosage optimization, diverse delivery strategies, and rigorous testing across various models of sepsis to ascertain efficacy and safety. Lastly, transitioning toward clinical research, it will be pivotal to assess ANXA1's viability as a biomarker for neuroinflammation and BBB integrity in patients with sepsis. Concurrently, human trials should be initiated to investigate ANXA1's therapeutic utility, meticulously examining its safety profile and effectiveness within a clinical context.

In conclusion, the clinical significance of these results lies in their potential to inform the development of targeted therapies for SAE and other neurological disorders involving BBB dysfunction. By elucidating the mechanisms underlying ANXA1's protective effects, we can work toward improving patient outcomes in conditions where BBB integrity is compromised.

## Ethics Statement

The protocols of animal studies were approved by the guidelines of Animal Care and Use Committee of Sichuan Provincial People's Hospital.

## Consent

All the authors agree to publish the manuscript.

## Conflicts of Interest

The authors declare no conflicts of interest.

## Supporting information


Data S1.

## Data Availability

The data of this study will be made available from the corresponding authors on request.
