# Supplementary material for: Annexin A1 Mitigates Blood–Brain Barrier Disruption in a Sepsis‐Associated Encephalopathy Model by Enhancing the Expression of Occludin and Zonula Occludens‐1 (ZO‐1)
Source: CNS Neurosci Ther. 2024 Dec 27;30(12):e70173. doi: 10.1111/cns.70173 (PMC11672249; doi:10.1111/cns.70173)

Full unedited gel/blot for Figure 1B

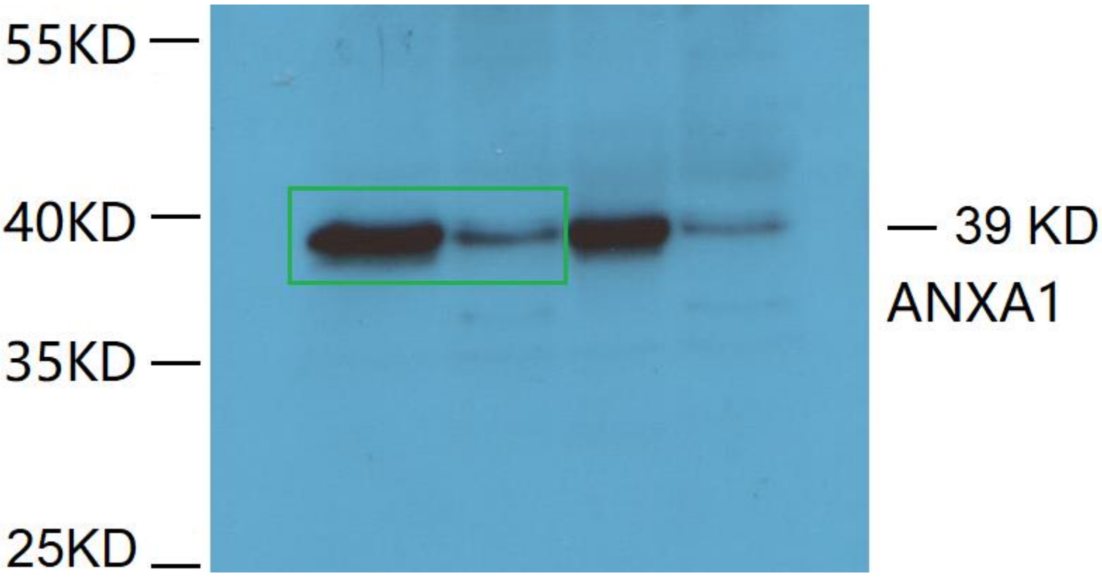

Full unedited gel/blot for Figure 1B

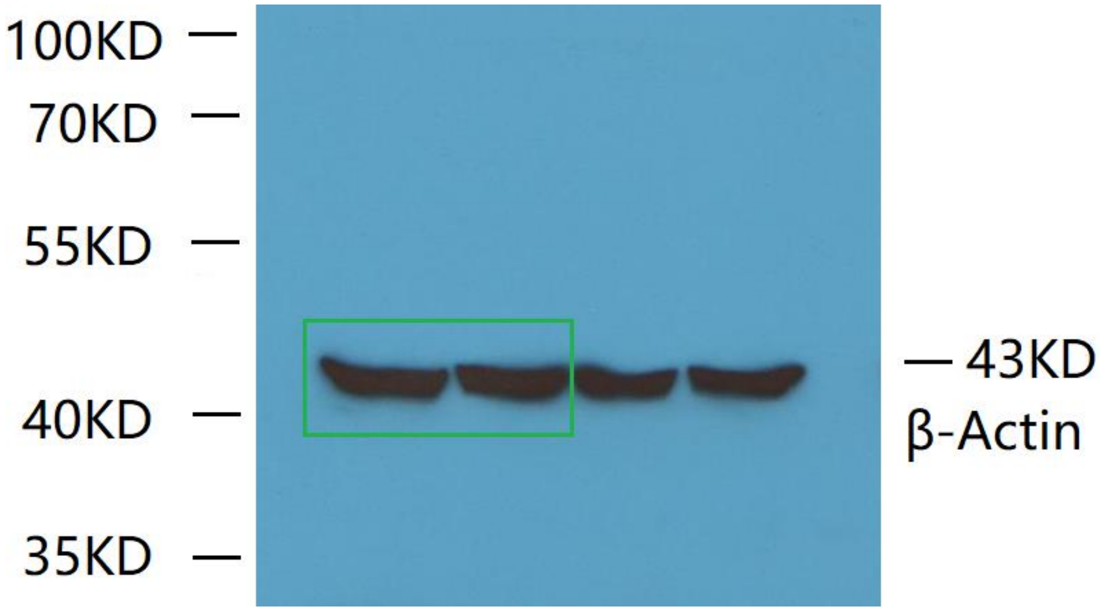

Full unedited gel/blot for Figure 7B

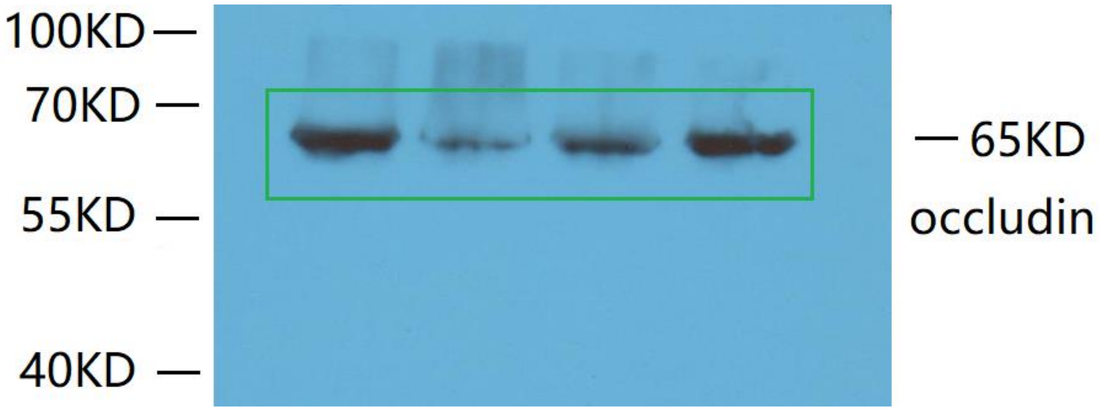

Full unedited gel/blot for Figure 7B

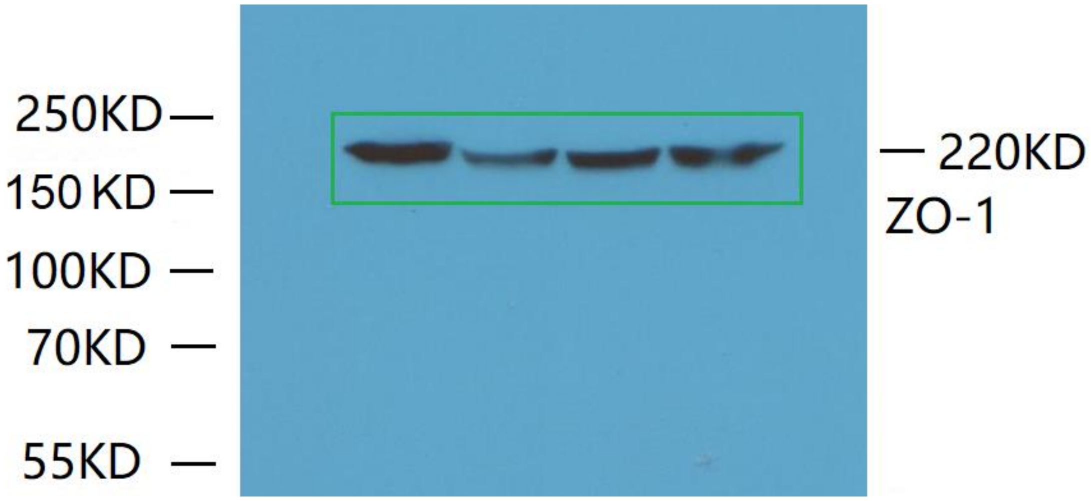

Full unedited gel/blot for Figure 7B

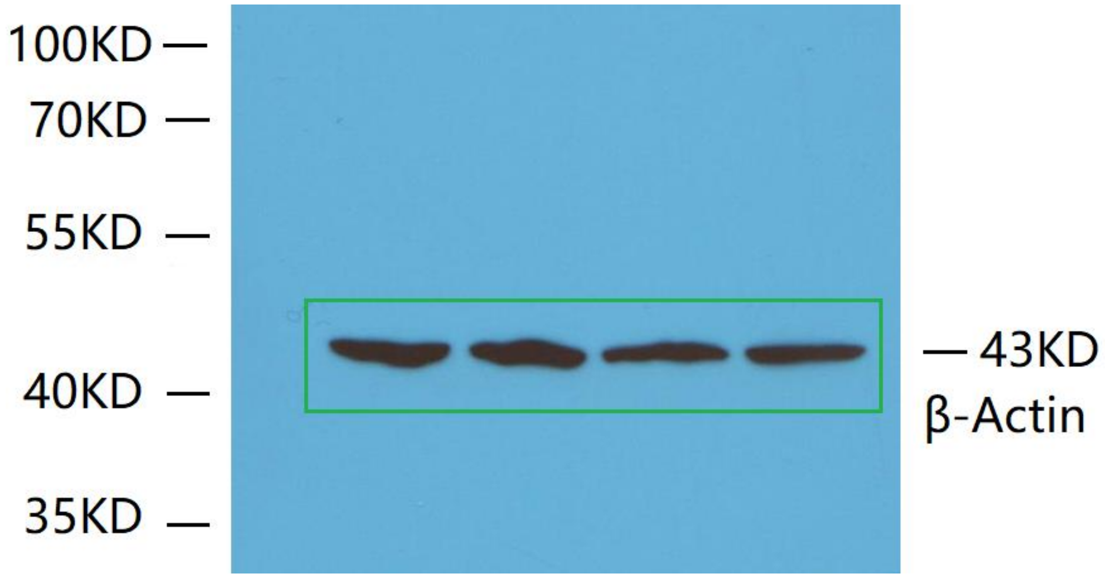

Full unedited gel/blot for Figure 8C

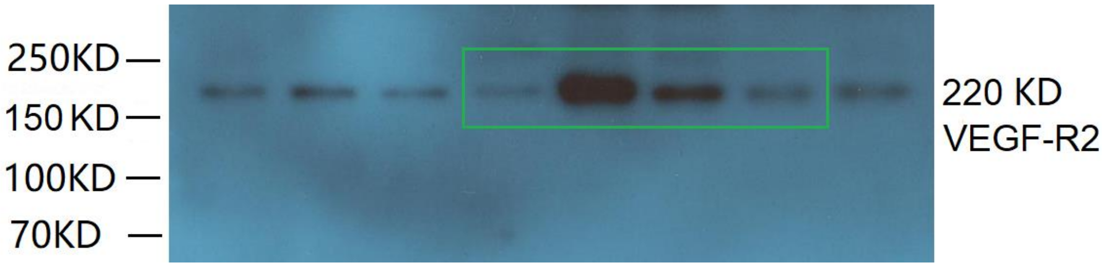

Full unedited gel/blot for Figure 8C

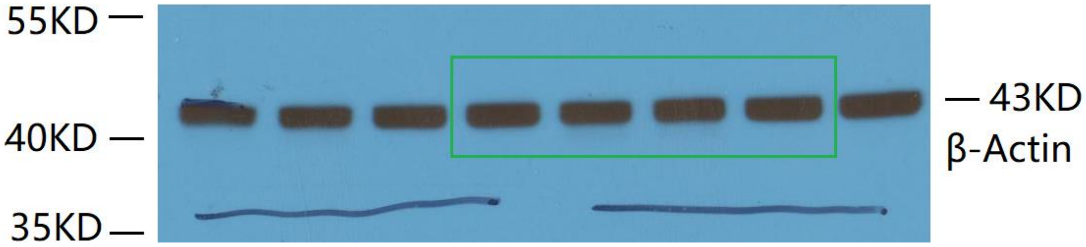

Full unedited gel/blot for Figure 9B

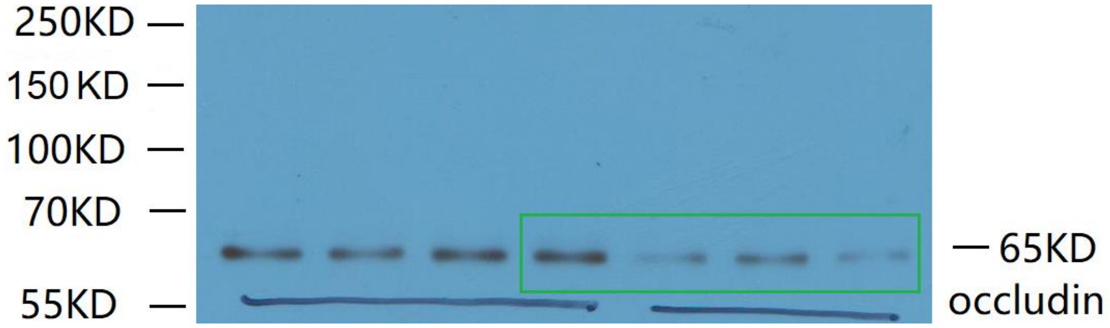

Full unedited gel/blot for Figure 9B

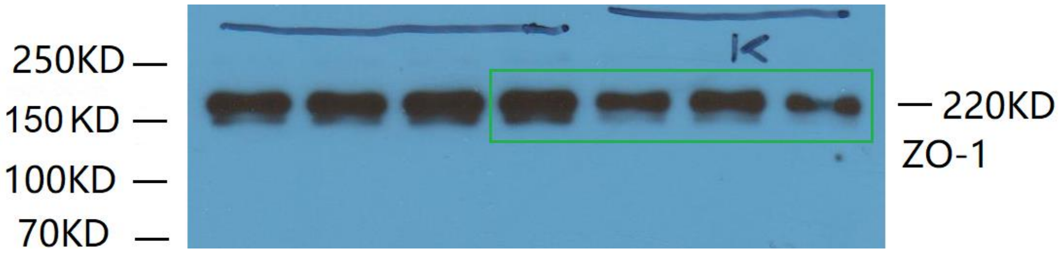

Full unedited gel/blot for Figure 9B

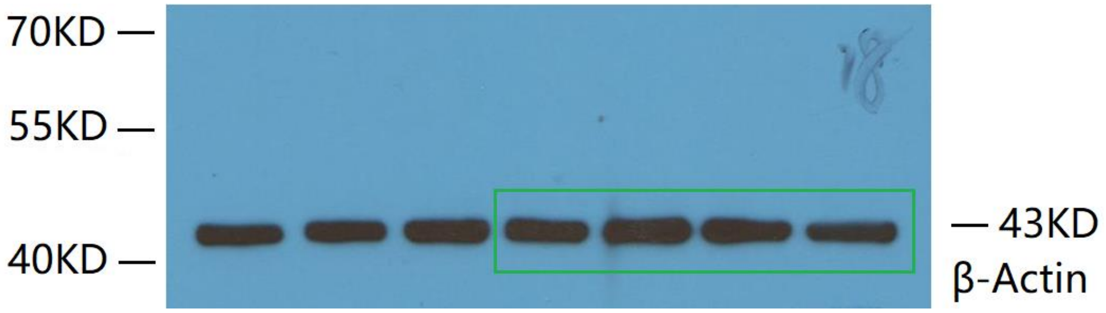

Supplement: Supplementary file 1 — Data S1. [file CNS-30-e70173-s001.pdf]
